# The influence of in vitro fitness defects on pneumococcal ability to colonize and to cause invasive disease

**DOI:** 10.1186/1471-2180-8-65

**Published:** 2008-04-18

**Authors:** Jenny Fernebro, Christel Blomberg, Eva Morfeldt, Hans Wolf-Watz, Staffan Normark, Birgitta Henriques Normark

**Affiliations:** 1 Swedish Institute for Infectious Disease Control, 171 82 Solna, Sweden; 2 Dep. of Microbiology, Tumor and Cell Biology, Karolinska Institutet, 171 77 Stockholm, Sweden; 3 Dep. of Molecular Biology, Umeå University, 901 87 Umeå, Sweden

## Abstract

**Background:**

*Streptococcus pneumoniae *is a genetically diverse major human pathogen, yet a common colonizer of the nasopharynx. Here we analyzed the influence of defects affecting *in vitro *growth rate, on the ability of *S. pneumoniae *to colonize and to cause invasive disease *in vivo*.

**Results:**

Of eleven different clinical isolates one serotype 14 carrier isolate showed a significantly longer generation time as compared to other isolates, and was severely attenuated in mice. To directly investigate the impact of growth rate on virulence, a panel of mutants in five non-essential housekeeping genes was constructed in the virulent TIGR4 background by insertion-deletion mutagenesis. Three of these mutants (*ychF*, *hemK *and *yebC*) were, to different degrees, growth defective, and showed a reduced invasiveness in an intranasal murine challenge model that correlated to their *in vitro *growth rate, but remained capable of colonizing the upper airways. The growth defect, as well as virulence defect of the *hemK *insertion-deletion mutant, was mediated by polarity effects on the downstream *yrdC *gene, encoding a probable chaperone in ribosome assembly.

**Conclusion:**

We conclude that large fitness defects are needed to completely prevent pneumococci from causing invasive disease after intranasal challenge. However, even severe growth defects still allow pneumococci to persistently colonize the upper airways.

## Background

*Streptococcus pneumoniae *is one of the major pathogens infecting humans worldwide. It is the most common cause of community-acquired bacterial pneumonia and childhood ear infections, but can also give rise to severe cases of meningitis and sepsis. It is estimated that 1–2 million people die from pneumococcal diseases every year [1].

The main pneumococcal virulence factor is the antiphagocytic capsular polysaccharide, depending on which pneumococci can be divided into at least 91 serotypes [2]. Other virulence factors described include cell wall components such as peptidoglycan, teichoic acid, lipoteichoic acid as well as cell surface proteins such as the choline-binding proteins [3,4] and the recently described pneumococcal pilus [5]. Signature-tagged mutagenesis (STM) has revealed a surprisingly large number of additional loci required for pneumococcal virulence in mice. Even though mutants in genes identified by STM are able to grow *in vitro*, it is not known to what extent these mutations cause fitness defects that might have an effect on virulence [6-8].

Despite causing severe diseases, pneumococci are also frequent colonizers of healthy individuals. 60–70% of children attending day-care centers may harbor these bacteria in their nasopharynx [9]. It is not fully understood which bacterial and host factors that contribute to the transition from carriage to disease. Invasive disease potential has been shown to differ between serotypes [10,11], but other genetic factors are also important for disease outcome in humans [10,12,13]. Previously we have shown in animal models that not only serotype, but also clonal type (based on multi locus sequence typing, MLST and pulsed-field gel electrophoresis, PFGE), affects clinical outcome [12].

Whether or not variation in growth rate among pneumococcal isolates affects virulence has not been investigated. Here we monitored the *in vitro *growth rate for a series of clinical pneumococcal isolates with different ability to grow in blood after intraperitoneal challenge of mice [12]. These isolates belong to different serotypes and different clonal types as determined by MLST. To further evaluate the impact of *in vitro *fitness on virulence, we generated mutants defective in non-essential genes belonging to the pneumococcal core genome and known to be universally present in both pathogenic and non-pathogenic bacteria [14] and therefore unlikely to represent bona fide virulence genes. We find that bacterial fitness defects, as monitored by bacterial growth rates *in vitro*, proportionally decreases pneumococcal virulence in animal models, without abolishing the ability to colonize the upper airways.

## Results

### In vitro growth rates of pneumococcal isolates and the relationship to colonization and virulence in mice

Previously, we studied a collection of clinical pneumococcal isolates of serotypes 1, 4, 6B, 7F, 14 and 19F belonging to different clonal types [12]. After intranasal challenge only TIGR4 and isolates of type 6B caused invasive disease. However, after intraperitoneal challenge both the median survival time and end-point survival rate differed between the isolates. TIGR4 and the two isolates of types 6B, 7F and 19F respectively were highly virulent after intraperitoneal infection, while isolates 1-B, 14-A, 1-A and 14-B were less virulent in decreasing order (Table 1) [12]. More than 90% of the mice survived >216 hours when inoculated with 14-B as compared to 50% with a challenge of 14-A.

**Table 1:** Bacterial strains used in this study

**Pneumococcal strains**	**Description**	**Source or reference**
**Clinical isolates**		
1-A (BHN31)	Serotype 1 invasive isolate of ST306	[12]
1-B (BHN32)	Serotype 1 invasive isolate of ST228	[12]
6B-A (BHN49)	Serotype 6B invasive isolate of ST138	[12]
6B-B (BHN50)	Serotype 6B carrier isolate of ST176	[12]
7F-A (BHN55)	Serotype 7F invasive isolate of ST191	[12]
7F-B (BHN56)	Serotype 7F carrier isolate of ST191	[12]
14-A (BHN78)	Serotype 14 invasive isolate of ST124	[12]
14-B (BHN83)	Serotype 14 carrier isolate of ST555	[12]
19F-A (BHN100)	Serotype 19F carrier isolate of ST162	[12]
19F-B (BHN97)	Serotype 19F carrier isolate of ST425	[12]
**Laboratory strains**		
TIGR4 (BHN126)	Wild-type strain of serotype 4 (Em^S ^Sm^S^)	[16]
TIGR4S (BHN160)	TIGR4 spontaneous streptomycin resistant mutant (Sm^R^)	This study
TIGR4Δ*ychF *(BHN168)	*ychF *(SP0004)::*erm *(Em^R^)	This study
TIGR4Δ*rluD *(BHN169)	*rluD *(SP0929)::*erm *(Em^R^)	This study
TIGR4Δ*smf *(BHN170)	*smf *(SP1266)::*erm *(Em^R^)	This study
TIGR4Δ*hemK *(BHN171)	*hemK *(SP1021)::*erm *(Em^R^)	This study
TIGR4Δ*yebC *(BHN172)	*yebC *(SP1922)::*erm *(Em^R^)	This study
TIGR4S*hemK*^- ^(BHN173)	Point mutations in *hemK *(SP1021) (Sm^R^)	This study
TIGR4S*yrdC*^- ^(BHN174)	Point mutations in *yrdC *(SP1022) (Sm^R^)	This study
TIGR4S*SP1023*^- ^(BHN175)	Point mutations in *SP1023 *(Sm^R^)	This study

**Figure 1:**
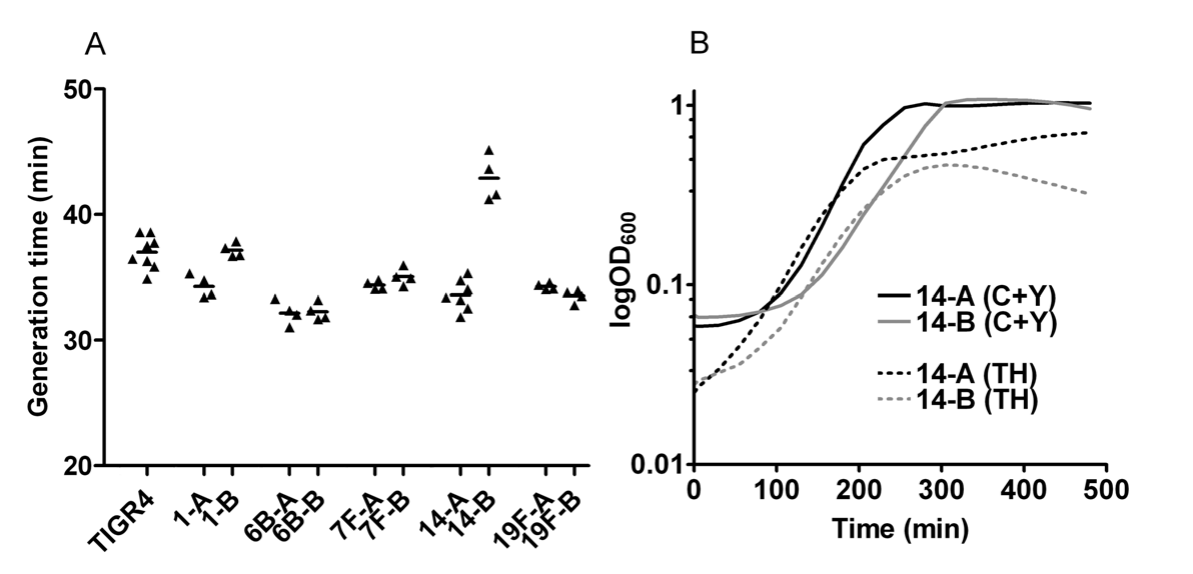
**A) Generation times in c+y of clinical isolates of serotypes 1 (1-A and 1-B), 4 (TIGR4), 6B (6B-A and 6B-B), 7F (7F-A and 7F-B), 14 (14-A and 14-B) and 19F (19F-A and 19F-B).B) **Growth curves of the two type 14 clinical isolates in c+y medium and TH medium.

To investigate whether the differences observed in survival *in vivo *could be correlated to differences in fitness, growth rate estimations and growth curves were made using a Bioscreen equipment (Figure 1A and 1B). All except one isolate showed generation times, in semisynthetic c+y medium, between 33 and 37 minutes. These differences in growth rates did not fully correspond to the abilities of the same isolates to grow in blood *in vivo *and to cause systemic disease [12]. Thus, isolate 1-B was more virulent than 1-A despite that the former isolate showed a slower *in vitro *growth rate. Nevertheless, it is interesting to note that the two 6B isolates showing the highest growth rates *in vitro *were the most virulent ones after intraperitoneal infection in mice. Only the Type14-B carrier isolate exhibited a significantly longer generation time (43 minutes) compared to all other isolates, which was in line with its low virulence potential in mice.

Comparative genomic hybridizations were next carried out on the isolates14-A and 14-B to identify gene content differences that could explain the fitness defect of the latter isolate. Microarrays were used, representing the full genomes of strains R6 [15] and TIGR4 [16]. The invasive serotype 14 isolate of ST124 (14-A) and the carrier isolate of ST555 (14-B) differed from one another at approximately 196 genes including some variable regions. R6-specific cluster 4 [17], C11* [17], C13* [17] and most of TIGR4-specific cluster 7 [17], also known as PPI-1, were present in 14-A but absent in 14-B. TIGR4-specific cluster 1 and 9 as well as C4*, C5* and part of R6-specific cluster 1 [17] were absent in 14-A, but present in 14-B. In addition, the two isolates differed from each other at 18 loci found to affect virulence by signature-tagged mutagenesis screen (Table 2). Six of these loci were absent in 14-B, seven were absent in 14-A and five had differences in sequence between the two isolates. One of the genes that varied in sequence was SP1018, which is included in the same operon as *hemK *(see below). Also, SP1019 of the *hemK *operon was missing in the slow-growing 14-B isolate, where SP1018 was directly followed by SP1020. SP1019 has previously been reported to be missing from some sequenced clinical isolates (see NCBI url in Availability and requirements section) [18].

**Table 2:** Microarray analysis of the clinical isolates 14-A and 14-B with respect to genes identified in STM [6-8]. Gene annotations are given according to TIGR4 [16].

**Gene**	**Description**	**Ref**	**14-A ST124**	**14-B ST555**
SP 0071	immunoglobulin A1 protease	[6, 8]	-	+
SP 0143	Hypothetical protein	[8]	-	+
SP 0265	bgla; 6 phospho beta glucosidase	[6]	-	+
SP 0396	mtlf; Mannitol specific enzyme IIA component	[6]	-	+
SP 0474	pts eii; Phosphotransferase system sugar specific EII component	[6]	-	+
SP 1321	v type sodium ATP synthase subunit K	[6]	-	+
SP 1328	sodium:solute symporter family protein	[6]	-	+
SP 0268	alkaline amylopullulanase putative	[6]	+^TIGR4^	+^R6^
SP 0629	conserved hypothetical protein	[7]	+^TIGR4^	+^R6^
SP 0933	pyrroline 5 carboxylate reductase	[7]	+^TIGR4^	+^R6^
SP 1018	tdk; Thymidine kinase	[6]	+^TIGR4^	+^R6^
SP 1029	RNA methyltransferase TrmA family	[6]	+^TIGR4^	+^R6^
SP 1343	ptrb; Protease II (oligopeptidase B)	[6]	+	-
SP 1344	Conserved hypothetical protein	[6]	+	-
SP 1433	Hypothetical protein	[6]	+	-
SP 1434	ABC transporter ATP binding/permease protein	[6]	+	-
SP 2095	Conserved hypothetical protein	[6]	+	-
SP 2164	pts eii; Phosphotransferase system sugar specific EII component	[6]	+	-

Note: Genes that are present are designated +, genes that are absent are designated -. In cases where sequence differences were found between the test isolates, subscripts indicate which reference strain the test strain hybridized to.

Even though we found large differences in the content of virulence and putative metabolic genes between the two type 14 isolates, we could not pinpoint one specific gene responsible for the observed defect in growth and virulence. Therefore we next studied isogenic mutants in conserved housekeeping genes in the pneumococcal genome.

### In vivo studies of isogenic mutants with different generation times

**Figure 2:**
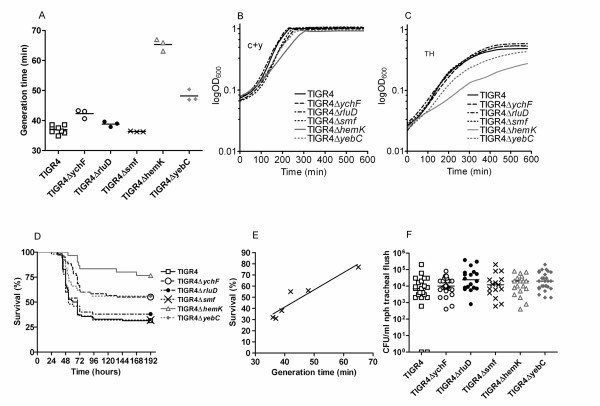
**Experiments with insertion-deletion mutants in the TIGR4 background.A) **Generation times in c+y medium. **B) **Growth curves in c+y medium. **C) **Growth curves in TH medium. **D) **Survival of mice after intranasal infection. **E) **Generation time of mutants versus survival of infected mice. **F) **Colonization of mice 8 days after intranasal infection.

To investigate the association between *in vitro *growth rate and *in vivo *ability to colonize and cause systemic disease insertion-deletion mutants were created in five genes in TIGR4, one of the most virulent strains in C57BL/6 mice in our collection. Microarray analysis of 40 pneumococcal isolates belonging to different serotypes and clones revealed that all carried the five genes, indicating that they belong to the pneumococcal core genome (data not shown). These highly conserved genes are present in all pathogenic as well as non-pathogenic bacterial species investigated so far [14], hence it is unlikely that they represent bona fide virulence genes. They were chosen since they appear to be house-keeping genes and gene inactivation would likely lead to fitness defects. They all have homologues in *E. coli*, hence we used their *E. coli *designations and named the mutants TIGR4Δ*ychF *(SP0004), TIGR4Δ*rluD *(SP0929), TIGR4Δ*smf *(SP1266), TIGR4Δ*hemK *(SP1021) and TIGR4Δ*yebC *(SP1922). When *in vitro *growth rates were measured in c+y semisynthetic medium, the *rluD *and *smf *mutants showed almost wild-type fitness with generation times of 39 and 36 minutes respectively, as compared to 37 minutes for the wild-type TIGR4 strain (p = 0.0596 and p = 0.4452) (Figure 2A and 2B). However, the insertion-deletion mutants of *ychF*, *hemK *and *yebC *all had reduced growth rates, 42, 65 and 48 minutes respectively (p = 0.0003, p < 0.0001 and p < 0.0001). The growth defect was especially pronounced for the *hemK *mutant, showing smaller colonies on blood agar plates. The growth differences for the mutant collection were also recorded in TH medium (Figure 2C). Also in this medium the *hemK *mutant showed the most pronounced growth rate defect and reached a lower density at stationary phase.

To mimic the natural route of infection, C57/Bl6 mice were challenged intranasally with the five insertion-deletion mutants respectively. All mutants gave rise to the same type of disease, but with different frequencies. For those mice where bacteria could be cultured from blood, health status rapidly and irreversibly deteriorated. At the time of sacrifice all mice with invasive pneumococcal infection had similar amounts of bacteria in the blood and lungs (5 × 10^7 ^CFU/ml and 1 × 10^5 ^CFU/mg respectively), irrespective of challenge strain. Thus, once bacteremia occurred, even the severely growth defective *hemK *mutant was not cleared from the blood stream. Three of the five mutants, TIGR4Δ*ychF*, TIGR4Δ*hemK *and TIGR4Δ*yebC*, gave a higher end-point survival than the wild-type TIGR4 strain with 55% (χ^2 ^= 10.25; p = 0.0014), 77% (χ^2 ^= 18.41; p < 0.0001) and 56% (χ^2 ^= 7.369; p = 0.0066) survival respectively by 192 hours, compared to 31% for TIGR4 (Figure 2D). In contrast, TIGR4Δ*rluD*, and TIGR4Δ*smf*, were not attenuated, with survival rates of 38% (χ^2 ^= 0.3529; p = 0.5525) and 32% (χ^2 ^= 0.0002264; p = 0.9880) respectively. There was a good relationship between end-point survival and *in vitro *growth rate (Figure 2E). The growth-defective mutants were capable of colonizing the mice to a similar extent 8 days post infection as TIGR4 (Figure 2F).

**Figure 3:**
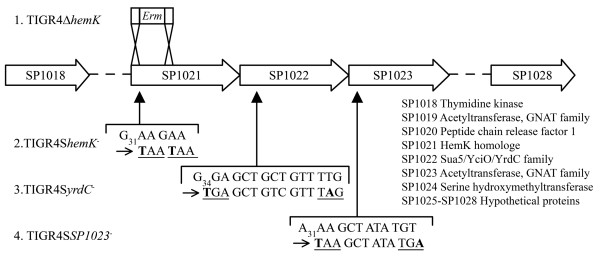
**The *hemK *operon and the mutants created in it.** TIGR4Δ*hemK *was made by insertion-deletion mutagenesis with an erythromycin cassette, while the other mutants contain in-frame stop codons (underlined) in the very beginning of the target genes. Alterations are shown in bold and the subscripts indicate the nucleotide number of the first alteration in each gene.

**Figure 4:**
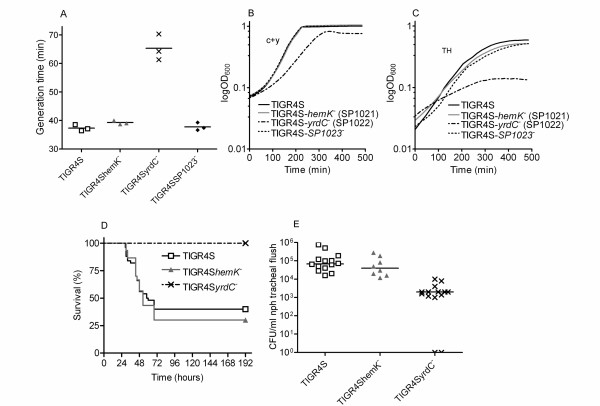
**Experiments with in-frame mutants in the TIGR4S background.A) **Generation times of strains grown in c+y medium. **B) **Growth curves in c+y medium. **C) **Growth curves in TH medium. **D) **Survival of mice after intranasal infection. **E) **Colonization of mice 8 days after intranasal infection.

Out of the five mutants, the growth defect was most pronounced for the *hemK *mutant. Since insertion-deletion mutagenesis is likely to give polar effects on downstream genes in the same operon, in-frame mutants were constructed in *hemK *and in the two genes directly downstream of *hemK *(SP1022 and SP1023) in order to pinpoint the responsible gene for the growth defect (Figure 3). Nucleotides were altered in the 5'-end of the open reading frames, introducing stop codons. The slow-growing insertion-deletion mutant of *hemK *gave smaller colonies than TIGR4 on blood agar plates. Of the in-frame mutants only the mutant in SP1022 (TIGR4S*yrdC*^-^) gave the same phenotype with small colonies, while the in-frame mutants of *hemK *(TIGR4S*hemK*^-^) and SP1023 (TIGR4S*SP1023*^-^) both showed wild-type colony size. The reduced colony size for TIGR4S*SP1022*^- ^corresponded to a reduced number of bacteria/colony (6 × 10^5 ^as compared to 2 × 10^6 ^for TIGR4S) after 20 hours of incubation on blood agar plates. Growth rate estimations with Bioscreen verified that only TIGR4S*yrdC*^- ^gave a longer generation time in c+y medium than TIGR4S, 65 minutes as compared to 37 minutes (p = 0.0005) (Figures 4A and 4B). The generation times for TIGR4S*hemK*^- ^and TIGR4S*SP1023*^- ^were 39 minutes (p = 0.0591) and 38 minutes (p= 0.6780) respectively. The same relative differences in *in vitro *growth rate for the different in frame mutants was also found after growth in TH-medium, with TIGR4S*hemK*^- ^and TIGR4S*SP1023*^-^exhibiting similar growth rate as TIGR4S and TIGR4S*yrdC*^- ^exhibiting a severe growth defect (Figure 4C).

Next, mice were challenged intranasally with TIGR4S, TIGR4S*hemK*^- ^or TIGR4S*yrdC*^- ^(Figure 4D). The survival rates with TIGR4S*hemK*^- ^and TIGR4S were 40% (χ^2 ^= 0.06550; p = 0.7980), with median survival time of 59 h and 68 h respectively. All mice survived after challenge with TIGR4S*yrdC*^- ^(χ^2 ^= 18.03; p < 0.0001). However, 12 out of 14 mice infected with this mutant remained colonized, as determined by CFU count from nasopharynealtracheal lavage at day 8 post-infection (Figure 4E), albeit at a lower level than those challenged with TIGR4S (median of 2 × 10^3 ^CFU/ml lavage versus 6.8 × 10^4 ^CFU/ml lavage (p = 0.0151)).

## Discussion

The pneumococcal genome consists of a core genome representing about 70% of the total gene content. The remaining 30% represents variable genes that may be present or absent in different isolates (unpublished data). Several of these variable genes are predicted to have metabolic functions, and therefore do not represent true virulence genes, even though *in vivo *screens using signature-tagged mutagenesis have identified a number of them as necessary to cause invasive disease in murine models [6-8]. Since *in vitro *fitness might be one explanation for differences found *in vivo *we aimed to determine whether *in vitro *fitness, as determined by *in vitro *growth rates, correlates to virulence *in vivo*. We used two clinical isolates each of serotypes 1, 6B, 7F, 14 and 19F with known virulence in an intraperitoneal mouse model [12] and compared growth rates with a Bioscreen apparatus. The two isolates of each serotype were chosen to be in most cases one carriage isolate and one invasive isolate that were genetically unrelated based on MLST profiles (Table 1). Differences in virulence had previously been detected between the two type 1 isolates, and an even more pronounced difference was found between the two type 14 isolates [12]. Major differences in *in vitro *growth rates between strains of the same serotype were detected only for type 14 isolates, suggesting *in vitro *growth to be an important parameter for the outcome *in vivo*. The two type 14 isolates were genetically unrelated by MLST (of ST124 and ST555 respectively) and microarray analysis, differing from each other at 18 loci found to effect virulence by STM screens (Table 2). Seven of these loci were absent in the more virulent 14-A isolate, while six were absent in 14-B. In five of the loci there were differences in sequence between the two isolates, with 14-A carrying genes equal to TIGR4, and 14-B having the R6 equivalents. Of the genes missing in slow-growing 14-B, at least two are predicted to be involved in transport functions (SP1434 and SP2164), but it is not known if defects in these genes affect growth.

For the two isolates of type 1, the differences in virulence could not be explained by differences in growth rates. Several features, besides growth fitness in a rich complex medium such as c+y, may influence the disease potential of a pneumococcal strain including the capsular type being expressed. This is also reflected by the fact that the differences seen in *in vitro *growth rate between the clinical isolates of different serotypes did not fully correspond to differences in mice virulence as monitored after intraperitoneal challenge. Ability to retrieve carbohydrates from complex glycoconjugates is likely important for pneumococcal ability to grow in different host environments [19]. Virulence-associated differences among clinical isolates in carbohydrate retrieval may only be revealed during *in vitro *growth using carbon sources mimicking the host environment, and was not further investigated in this paper. Instead we attempted to correlate the relationship between *in vitro *growth rate and *in vivo *virulence in mice creating a panel of mutants in the TIGR4 background deficient in highly conserved non-essential house keeping genes that were unlikely to affect carbohydrate metabolism of *Streptococcus pneumoniae*. TIGR4 was used since it is fully sequenced and since it is the most virulent strain in our collection after intranasal challenge.

The five candidate genes in the pneumococcal core genome were chosen based on a previous publication by Garbom et al. [14]. Garbom et al. showed that these genes are needed for full virulence of *Yersinia in vivo*, but they have only been considered as housekeeping genes in pneumococci. Their functions have been predicted either in *E. coli *or in other species, except for *yebC*. The *E. coli *YchF protein belongs to a group of 11 universally conserved GTPases [20]. Studies in *Haemophilus influenzae *have shown that YchF contains binding sites for both GTP and double-stranded nucleic acid, indicating that YchF may be part of a nucleoprotein complex and function as a GTP-dependant translation factor [21]. *rluD *encodes a pseudouridine synthase in *E. coli *which is responsible for the production of 23S rRNA pseudouridines 1911, 1915 and 1917 [22]. This protein is non-essential in *E. coli*, but affects growth [22]. SP0927, which is transcribed upstream of the pneumococcal homolog to *rluD *has been identified as essential for lung infection in mice in a signature mutagenesis screen (STM) [6]. *smf *has a homologue in *H. pylori*, *B. subtilis*, and *Streptococcus pneumoniae*, named *dprA *(SP1266), and is involved in natural competence [23-25]. It has only been linked to virulence in *Yersinia *and *N. meningitidis *[14,26]. The pneumococcal DprA protein is proposed to play a role in the late stage of transformation, by binding to incoming single stranded DNA and bringing it to the RecA recombinase. Finally, *hemK *in *E. coli *encodes an *N*(5)-glutamine methyltransferase that modifies peptide release factors [27].

Insertion-deletion mutants (potentially creating polar effects on transcriptionally downstream genes) were constructed for all five genes, *ychF*, *rluD*, *smf*, *hemK *and *yebC *in the TIGR4 background. Defects in *in vitro *growth were observed for mutants in *ychF*, *hemK *and *yebC *respectively, where the HemK-deficient mutant was most severely attenuated with an 80% longer generation time as compared to the wild-type TIGR4. In an intranasal mouse model of infection, mutations that did not affect *in vitro *fitness, i.e. *rluD *and *smf*, did not influence virulence, while all mutants with growth defects (*ychF*, *hemK *and *yebC*) were also attenuated in virulence. The reduction of virulence correlated to the extent of growth defect observed. Hence, the *hemK *mutant was most attenuated, but still capable of causing severe invasive disease in 27% of the animals. Long-term colonization of the upper airways was not affected by any of the mutants. Thus, fitness defects reducing growth rate *in vitro *did not affect non-symptomatic carriage, but affected the likelihood of transition from carriage to invasive disease. However, once invasive disease occurred there was no apparent effect of a fitness defect on the progression of invasive disease.

For the mutants in *ychF *(SP0004) and *yebC *(SP1922), we did not investigate possible effects on neighboring genes. The pneumococcal homologue to *ychF *was reported as potentially essential by Thanassi et al., but Song et al. showed that this effect was due to polarity [28,29]. Instead Song et al. identified SPR0003, the R6 equivalent of SP0003, which is probably transcribed together with *ychF*, as essential. Therefore, it is possible that the phenotype of our *ychF *insertion-deletion mutant is due to effects on SP0003 or polar effects on another essential gene SP0005 [29] encoding a putative peptidyl-tRNA hydrolase. Also in the case of *yebC *(SP1922), interesting genes can be found in close proximity. About 1 kb downstream of *yebC *is *ply*, encoding the well-known pneumococcal virulence factor pneumolysin [30]. These genes are however not believed to be co-transcribed, since the *ply *gene is transcribed from its own promoter [31].

In *S. pneumoniae, hemK *is the fourth gene out of eleven in an operon that has previously been reported to be associated with virulence [32,33]. Two groups have reported the effects of mutations in *hemK *or the *hemK *operon in *S. pneumoniae*. While Marra et al. observed a significant effect on virulence, Orihuela et al. did not observe any attenuation [32,33]. Hava and Camilli identified SP1023 (two genes downstream of *hemK*) as essential for lung infection in mice using STM, but did not study the mutant further [6]. To investigate which gene(s) in this operon was attributed to the observed defect in growth and virulence, we constructed in-frame mutants for *hemK *and the two genes downstream of *hemK*, SP1022 and SP1023. The HemK-deficient insertion-deletion mutant showed smaller colonies on blood agar plates, hence we studied the in-frame mutants for this phenotype and found it only in the SP1022 mutant. This mutant also had a considerably longer generation time in c+y as well as in TH medium compared to the parental strain, which was not the case for the other in-frame mutants. Also, the SP1022 mutant was completely non-virulent *in vivo*, while the non-polar *hemK *in frame mutant gave wild-type virulence. In this set of experiments all derivatives used carried a mutation in *rpsL *giving streptomycin resistance. The lower virulence observed for TIGR4S as compared to TIGR4 is likely due to the fitness cost of this *rpsL *mutation as previously shown for *Salmonella typhimurium *[34]. The complete attenuation of the in-frame mutation in SP1022 likely reflects the added fitness defect contributed by the *rpsL *mutation. SP1022 is a homologue to *yrdC *in *E. coli*, which was recently reported to be responsible for ribosome maturation [35] and mutations in this gene resulted in severe defects in growth. Non-ribosomal factors in ribosome subunit assembly such as YrdC are emerging targets for new antibacterial drugs [36]. We cannot rule out that also SP1023 (as well as other genes downstream in the operon) may be involved in virulence, without affecting *in vitro *fitness. SP1023 was picked up in an STM screen by Hava and Camilli [6]. The method used by Hava and Camilli to construct the mutant library may have lead to effects on the expression from neighboring genes within the operons of the targeted genes. Hence, it is possible that the phenotype reported for SP01023 is due to effects on *yrdC *(SP1022) expression.

## Conclusion

Here we found support both among our clinical isolates studied as well as among the constructed mutants that *in vitro *growth affects invasiveness *in vivo*. However, all TIGR4 mutants with reduced growth rate remained capable of causing severe invasive disease in a fraction of mice, and were still able to colonize the upper airways to the same level as the wild-type strain. Also, the slowly growing ST555 isolate of serotype 14 (14-B) was isolated from the nasopharynx of a human being. Our results therefore suggest that pneumococci with metabolic or other fitness defects may prevail in the carrier population, and potentially also cause invasive disease. Such strains could possibly act as reservoir for the build up of better-fit strains that also are more capable of generating invasive disease. It is surprising that so many pneumococcal loci have been shown, by signature-tagged mutagenesis, to be required for virulence in mice [6-8]. Revisiting this collection of mutants, asking which ones also exhibit *in vitro *fitness defects, may significantly reduce the number of pneumococcal loci that are directly involved in overcoming host defenses during invasive disease. Furthermore, it may be fruitful to identify conserved surface-exposed proteins that provide a growth defect when inactivated and use these proteins as drug targets or as recently reported as vaccine targets [37].

## Methods

### Bacterial strains used

The bacterial strains used in this study are listed in Table 1. Bacteria were grown on blood agar plates over night, were resuspended in glucose medium supplemented with 10% horse serum and inoculated 1:10 in semisynthetic c+y medium [38], and grown until appropriate OD_620_.

### In vitro fitness as determined by monitoring growth

To determine fitness of clinical isolates and mutants, bacterial growth in semisynthetic c+y medium [38] was monitored in Bioscreen (Labsystems, Finland). Measurements were made at OD_600 _every fifth minute for sixteen hours and the experiment was repeated three times. Growth curves were made and generation times were calculated from the slope of the growth curves at mid-log phase. Growth curves were also made based on growth in TH medium.

### Microarray analysis

Comparative genomic hybridizations were carried out on two type 14 clinical isolates using a reference design as previously described [39]. Four replicate experiments, including dye-swap, were performed. Oligonucleotides were based on predicted open reading frames of the two sequenced strains R6 [15] and TIGR4 [16]. The data were analyzed using Genepix pro 6.0 and the R Project for Statistical Computing (see Availability and requirements section for url) as previously described [39]. For statistical analysis we used a Bayesian linear model [40] and the Holm multiple testing correction to adjust individual p-values. This method was used to compare the isolates to the reference strains. Genes were considered absent if they had a p-value less then 0.01 within M-value less than -1 and present if they had an M-value of more than -0.8. Genes were considered to have sequence differences when one isolate bound to the oligonucleotide corresponding to R6 and the other to the oligonucleotide corresponding to TIGR4.

### Housekeeping genes selected for further analysis

To study the effect of growth on virulence, five universally conserved housekeeping genes shown to affect virulence in *Yersinia *[14] were selected. All five genes have homologues in *E. coli*, designated *ychF*, *rluD*, *smf*, *hemK *and *yebC*. The respective pneumococcal equivalents in the sequenced TIGR4 and R6 strains are SP0004/SPR0004, SP0929/SPR0830, SP1266/SPR1144 (*dprA*), SP1021/SPR0925 and SP1922/SPR1738 respectively [15,16].

### Construction of mutants using insertion-deletion mutagenesis

Flanking regions of target genes were amplified from TIGR4 using specific primers (Appendix) giving either *Apa*I or *Bam*HI restriction sites. The erythromycin cassette from pVA838 [41] was amplified with *Apa*I and *Bam*HI termini (Appendix). All fragments were digested and ligations were performed with upstream, downstream and erythromycin fragments. TIGR4 was transformed with the ligation products, with selection for Em^R^. Transformations were made by adding DNA and CSP-2 [42] to the recipient pneumococcal strain at OD_620 _= 0.1, followed by incubation on ice, at 31°C and finally at 37°C. Transformants were verified by PCR with control primers (Appendix).

### Construction of stop codon mutants (Janus)

Stop codon mutants were constructed for *hemK *and the two genes downstream of *hemK *(SP1022 and SP1023). These were produced using the Janus cassette [43] with both positive (kanamycin) and negative (streptomycin) selection. The wild-type strain was a streptomycin-resistant TIGR4 mutant, TIGR4S, spontaneously obtained through selection on streptomycin-containing agar plates and containing a substitution (K56 → R56) in the *rpsL *gene, previously reported to cause streptomycin resistance [44]. A similar construct as for the insertion-deletion mutants was made, but with the Janus cassette instead of the erythromycin cassette, using primers DAM406 and DAM351 [43]. This ligation mix was first transformed into TIGR4 with selection on kanamycin-containing plates. PCR was run over the full construct and this fragment in turn was transformed into TIGR4S. Positive clones of TIGR4S were then transformed with PCR products containing stop codons. These were introduced about ten amino acids downstream of the initial ATG.

### In vivo studies

5–8 weeks old C57BL/6 mice were inoculated intranasally with 5 × 10^6 ^CFU. Mice were monitored for 8 days to assess the health status by clinical scoring and blood samples taken daily. The mice health status was monitored according to the following scores: 0 = healthy, 1 = piloerection, 2 = reduced motility, 3 = more pronounced reduced motility, 4 = 1, 2, 3 more pronounced and 5 = moribund. Mice were sacrificed when they reached score ≥ 3. Surviving mice were killed on day 8 and a nasopharyngealtracheal flush was performed to monitor colonization. All experiments were repeated at least three times. The studies were approved by The Ethical Committee for Animal Experiments in Stockholm. All animals were kept with a 12-hours light/dark cycle and had access to standard food and tap water ad libitum.

### Statistical analysis

For survival studies, differences were analysed by the Kaplan-Meier analysis log-rank test. The generation times were analysed using the student *t*-test (two-tailed).*P *< 0.05 was considered significant.

## Availability and requirements

NCBI: 

R Project for Statistical Computing: 

## Authors' contributions

JF and CB carried out the laboratory experiments. JF, CB, EM, HWW, SN and BHN designed the study and wrote the manuscript. All authors read and approved the final manuscript.

## Appendix

See Table 3.

**Table 3:** Primers used in this study

**Primer**	**Sequence**
YchF-up5'	AAGTACACAAGAGAGTCGTCCGAT
YchF-up3'	TTGGGCCCTCTCCGTTTTCATTTCAATCCC
YchF-down5'	TTGGATCCAATGGTGTCAATTAGGTTGGAA
YchF-down3'	TGATCATATTCTTCACCAACTGAGA
YchF-C1	GAAATTTCAGCAAGCACTCC
YchF-C2	ACTGATGGATAAACGCGTGT
RluD-up5'	TTCTTGCCACCAACTATTACGGC
RluD-up3'	TTTGGGCCCAGCCTTATCCAAACGCAGAC
RluD-down5'	TTTGGATCCAATTTAAAGCAGATATCCCAGAGA
RluD-down3'	ATGTTGAAATATTGACAAAACCGTCT
RluD-C1	TCAGTATGCCAATCCTGAAG
RluD-C2	CTTGATACCACCAGTTCCC
DprA-up5'	TTGTCCTATCTTGTGATTGTGCTC
DprA-up3'	TTTGGGCCCTTCTACTCATCTATCTATTCGT
DprA-down5'	TTTGGATCCAGTGGGCAAGATGTTCTTGC
DprA-down3'	CGATAGAGGCGATAAGCATGGC
DprA-C1	GAGTGAAATCGAAGCCCTC
DprA-C2	AATCACGGGAGTCGAATCC
HemK-up5'	CAACTACAAGTTGTAGAAGACCGT
HemK-up3'	TTTGGGCCCTGAGCTAATTTCATTATTTGTTTA
HemK-down5'	TTTGGATCCTTGGTCAAGATAGGATGGTTGT
HemK-down3'	TCTCCTTTATTGTGTGACTAGTCC
HemK-C1	GAGAATGAAGTAGTAGCAATC
HemK-C2	CAGAGATCAGCATCATATGC
HemK-point1	CAACTCTT**A**TT**A**AAAATTTGAAAATAATTGAGC (point mutations in bold)
HemK-point2	TTTCAAATTTT**T**AA**T**AAGAGTTGATAAGACAAGGAG (point mutations in bold)
YebC-up5'	TTTGCCACTAGTGCGTAAGCGG
YebC-up3'	TTGGGCCCTACGTCCCATTAGGAATCTCC
YebC-down5'	TTGGATCCACCTATAGAACATGATCCTAAGTG
YebC-down3'	CTTCCCAATAGCCCAGATAGCC
YebC-C1	TTAGAGAGTGTACCGGGC
YebC-C2	AGGACTGTTTTGGAAAGGC
ErmApa	TTTTTGGGCCCTTCGTGTTCGTGCTGACTTGC
ErmBam	TTTTTGGATCCGATGTTGCTGATTAAGACGAGC
SP1022-point1	TCTGTAGGC**T**AAACGACAGCTC**A**ACCATTT (point mutations in bold)
SP1022-point2	AAATGGT**T**GAGCTGTCGTTT**A**GCCTACAGA (point mutations in bold)
SP1022-up5'	TTTGCCACTAGTGCGTAAGCGG
SP1022-up3'	TTGGGCCCTACGTCCCATTAGGAATCTCC
SP1022-down5'	TTGGATCCACCTATAGAACATGATCCTAAGTG
SP1022-down3'	CTTCCCAATAGCCCAGATAGCC
SP1022-C1	TCAGATTGCTCAAGATGAGC
SP1022-C2	TTCCACCTGAAATAATACGGA
SP1023-point1	GGTTGATGTC**T**CATATAGCTT**A**CACATCAG (point mutations in bold)
SP1023-point2	CTGATGTG**T**AAGCTATATG**A**GACATCAACC (point mutations in bold)
SP1023-up5'	AGATTGGTCAGTGACGGCA
SP1023-up3'	TTGGGCCCAGTTTCTCTTAAATCTCTTAGC
SP1023-down5'	TTGGATCCAGAAACGGTTTATTCGCATCT
SP1023-down3'	TTCCACCTGAAATAATACGGA
SP1023-C1	TAGTGGAGCTATTGCTCTC
SP1023-C2	ATCCAACAAGTTTTGAGCAAC
